# Interconvertible Lac Repressor–DNA Loops Revealed by Single-Molecule Experiments

**DOI:** 10.1371/journal.pbio.0060232

**Published:** 2008-09-30

**Authors:** Oi Kwan Wong, Martin Guthold, Dorothy A Erie, Jeff Gelles

**Affiliations:** 1 Department of Biochemistry, Brandeis University, Waltham, Massachusetts, United States of America; 2 Department of Physics, Wake Forest University, Winston-Salem, North Carolina, United States of America; 3 Department of Chemistry and Curriculum Applied and Materials Sciences, University of North Carolina at Chapel Hill, Chapel Hill, North Carolina, United States of America; Stanford University, United States of America

## Abstract

At many promoters, transcription is regulated by simultaneous binding of a protein to multiple sites on DNA, but the structures and dynamics of such transcription factor-mediated DNA loops are poorly understood. We directly examined in vitro loop formation mediated by Escherichia coli lactose repressor using single-molecule structural and kinetics methods. Small (∼150 bp) loops form quickly and stably, even with out-of-phase operator spacings. Unexpectedly, repeated spontaneous transitions between two distinct loop structures were observed in individual protein–DNA complexes. The results imply a dynamic equilibrium between a novel loop structure with the repressor in its crystallographic “V” conformation and a second structure with a more extended linear repressor conformation that substantially lessens the DNA bending strain. The ability to switch between different loop structures may help to explain how robust transcription regulation is maintained even though the mechanical work required to form a loop may change substantially with metabolic conditions.

## Author Summary

Some proteins that regulate DNA transcription do so by binding simultaneously to two separated sites on the DNA molecule, forming a DNA loop. Although such loops are common, many of their features are poorly characterized. Of particular interest is the question of how some proteins accommodate the formation of loops of different sizes, particularly when the loops are small and thus require strong bending (and, in some cases, twisting) of the DNA to form. We observed the shape and behavior of individual DNA molecules bent into tight loops by Lac repressor, a transcription-regulating protein from the bacterium Escherichia coli. Loops were formed in DNA molecules with repressor-binding sites on opposite faces of the DNA double helix almost as readily as in those with sites on the same side, suggesting that the repressor is highly flexible. The DNA can switch back and forth between a tighter and a looser loop structure “on the fly” during the lifetime of a single loop, further evidence that Lac repressor is capable of adopting different shapes that may serve to minimize DNA bending or twisting in loops. The ability of the repressor to readily switch between different loop shapes may allow it to maintain effective control of transcription across situations in which the difficulty of bending or twisting DNA changes substantially.

## Abstract 3

A large-scale conformational change in a transcription factor protein allows DNA loops to dynamically switch between alternative conformations that may contribute to robust transcription regulation.

## Introduction

DNA looping, in which a protein or protein complex interacts simultaneously with two separated sites on a DNA molecule, is a recurring theme in transcription regulation [1]. A prototypical example is transcription initiation at the *E. coli lacZYA* promoter, which is modulated through DNA looping by the lactose repressor. The promoter vicinity includes three operator sites: a primary operator (*O*_1_) located 11 bp downstream from the *lacZ* transcription start site, and two auxiliary operators (*O*_2_ and *O*_3_) with lower affinities for the repressor located 401 bp downstream and 92 bp upstream of *O*_1_, respectively (see review [2]). Repressor binding to *O*_1_ blocks transcription from the *lacZYA* promoter. Nevertheless, the presence of *O*_2_ and *O*_3_ is indispensable for complete transcriptional repression in wild-type bacterial strains because the repressor loops DNA by binding simultaneously to *O*_1_ and *O*_2_ or *O*_3_ [3–6], and such looping enhances repression by increasing the occupancy of *O*_1_ by repressor [5,7].

In many transcription factors that function at least in part by DNA looping (for example, the lambda, ara, and gal repressors [8–10]), the protein complex interacts with two binding sites displayed on the same face of the double helix. Both in vitro and in vivo, these systems display a characteristic dependence of repression on interoperator spacing, with strong repression when operators are separated by an integer number of helical repeats (“in phase”), and repression weak or absent when an additional half turn of the helix is added (“out of phase”) [9,11–14]. The reduced repression with out-of-phase operators is consistent with simple models of DNA elasticity, which predict a substantial energy cost to twist a short interoperator DNA segment by a half turn. In contrast, the effects of operator phasing on DNA looping by Lac repressor are in general weaker than those seen with other well-characterized bacterial repressors. Also, there is strong evidence from studies in vitro ([15,16] and references therein) and in vivo [17] that stable looped repressor–DNA complexes can form with operator spacings as small as or smaller than the 92-bp *O*_1_–*O*_3_ spacing. Even spacing the operators so that they are positioned on opposite sides of the double helix only 14.5 and 15.5 turns apart, so that substantial DNA twisting and bending may be required to close the loop, allows formation of putatively looped species, apparently with only a modest reduction in stability relative to similarly sized in-phase loops [15]. Out-of-phase operator spacings of similar size also give levels of repression in vivo consistent with looping [18].

No direct determinations of the structures of small Lac repressor–DNA looped complexes are available. The availability of crystallographic structures for the repressor alone and in complex with two DNA oligonucleotides [19,20], together with studies of the thermodynamic and kinetic stabilities of Lac repressor–DNA looped complexes in vitro [7,21–25], have led to the proposal of a variety of different structural models for looped protein–DNA complexes [1,19,20,24,26–28]. Most of these models are based on the crystallographic repressor–oligonucleotide model and a smoothly bent interoperator DNA segment. However, the tightly bent or strongly twisted interoperator DNA in these models is predicted to be highly energetically unfavorable based on simple worm-like-chain (WLC) models of DNA mechanics [29]. One proposal is that the energy of bent or twisted loops is reduced by introducing a kink into the DNA [30–33], but this idea is controversial [34]. It has also been suggested that large-scale alterations in repressor structure might help to accommodate small loops [19,20,26,28,35–39]. Nevertheless, neither the number of looped species that can form with small operator spacings nor their structure(s) nor their dynamics is known with any certainty.

**Figure 1:**
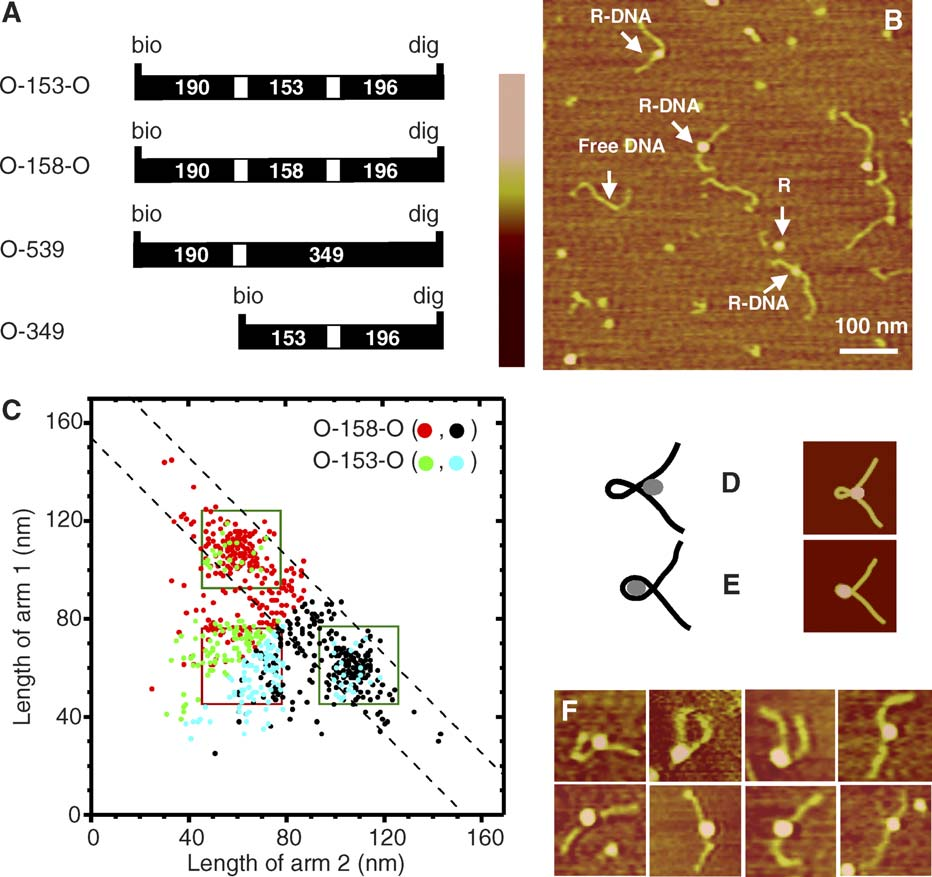
Atomic Force Microscopy of Lac Repressor–DNA Complexes (A) Schematic structures of biotin (bio)- and digoxigenin (dig)-labeled DNA constructs with one (O-539 and O-349) or two (O-153-O and O-158-O) ideal *lac* operator sequences (white bars) [82]. Numerals are DNA segment lengths (base pairs) measured from the center(s) of the operator(s). (B) AFM image of molecules adsorbed to a mica surface (see Materials and Methods) from a mixture of repressor and di-operator DNA O-153-O (183-nm contour length). R, repressor; R-DNA, repressor–DNA complex. The color scale corresponds to a 3-nm range of heights. (C) Scatter plot of the DNA arm contour lengths in repressor–DNA complexes. Measurements were made on images of complexes (*n* = 364) that had a single repressor molecule with two well-resolved protruding DNA arms; complexes with only one arm or with an arm that was too short for accurate measurement were excluded. In this graph, the data from each complex are plotted twice: the length of the longer arm (on the vertical axis) is plotted against the length of the shorter arm (horizontal axis); the same data are then replotted in a different color with the two axes interchanged. This produces a representation of the data that is symmetrical across a line (not shown) extending from the lower left to upper right corner. Data from both O-158-O (red and black) and O-153-O (green and blue) containing samples are included. The region between the dashed lines is that in which the sum of the arms lengths is 169 ± 16 nm, the image contour length mean ± 2 × S.D. measured for the uncomplexed DNAs. Green and red squares enclose the ranges of arm lengths predicted for operator-specific linear and looped repressor–DNA complexes, respectively. (D and E) Left: projection images of two alternative three-dimensional structural models of looped complexes between Lac repressor (gray) and DNA (black) with two operators spaced approximately 50 nm apart. Right: the corresponding AFM images produced by numerical simulation of the AFM imaging process (see Materials and Methods). (F) Representative images (120 × 120 nm) of looped complexes with O-158-O or O-153-O DNA.

To further characterize the reactions and structural properties of small Lac repressor–DNA loops, we employed single-molecule kinetic and imaging techniques to examine loop formation from two different di-operator DNA constructs, O-158-O and O-153-O, that have interoperator separation of 15 and 14.5 helical turns, respectively (Figure 1A). These two constructs are identical in sequence, except for the 5-bp insertion in O-158-O. To characterize the conformation of the Lac repressor–DNA complexes formed with these DNAs, we used atomic force microscopy (AFM) to directly visualize individual complexes. To follow loop formation and breakdown in single DNA molecules, we used tethered particle motion (TPM) single-molecule light microscopy [40,41], which can directly observe DNA looping events mediated by Lac repressor [23,42,43] and other proteins [44–49] by monitoring the extent of Brownian motion of a microscopic bead attached to a single surface-immobilized DNA molecule. AFM images of individual surface-immobilized looped complexes revealed that the DNA is wrapped around Lac repressor, and analysis of loop formation and breakdown by TPM demonstrated the presence of multiple looped structures in equilibrium with one another (in the case of O-158-O), defined the kinetic mechanisms of looping, and provided additional structural information. The data imply novel structures for some of the loops and suggest that the Lac repressor–DNA system is capable of adopting multiple polymorphic structures that help to lessen the mechanical strains inherent in forming small loops.

## Results

### Lac Repressor Forms Small Loops, Even in DNAs with Out-of-Phase Operators

AFM is an excellent method for examining protein-induced conformational changes in DNA because it allows direct visualization of individual protein–DNA complexes. It has been used to assess DNA looping by other proteins [50–56]. We used AFM to examine the complexes of Lac repressor with either of the two di-operator DNAs. A representative image of O-153-O DNA deposited in the presence of Lac repressor is shown in Figure 1B. Both free DNA and DNA with Lac repressor bound can be seen. Because we know the positions of the operator binding sites in the DNA, we can determine whether Lac repressor is bound to a single operator site, to two operator sites simultaneously (looping the DNA), or to nonspecific sites by measuring the contour length of the DNA and the distance of Lac repressor from the end of the DNA. The contour length (see Materials and Methods) measured for images of DNA molecules without repressor molecules bound is 169 ± 8 nm (mean ± standard deviation [S.D.]; *n* = 105), close to the expected contour lengths of the O-153-O and O-158-O DNAs (183 and 185 nm, respectively). This confirms that the image analysis method reliably reports DNA contour length.

For DNA with protein bound, the contour length of each DNA arm was measured from the center of the Lac repressor protein. The two operator sites are each roughly the same distance from the DNA ends. Thus, if repressor is bound to either site alone, it will have a long arm and a short arm, with the sum of the arm lengths equal to the free DNA length. Alternatively, if repressor is simultaneously bound to both sites, both arms should be of approximately equal length and the sum of their lengths should be approximately 150 bp shorter than that of free DNA. Figure 1C shows a plot of the contour length of the longer arm versus the shorter arm for all DNAs with a single Lac repressor bound. For a little more than half (205 of 364) of the complexes, the sum of the lengths of the two arms falls within two S.D. of the length of free DNA (the region delineated by black dashed lines in Figure 1C), consistent with Lac repressor being bound to linear unlooped DNA. Of these 205 complexes, most (136) fall into a distinct, small cluster with the combination of longer- and shorter-arm contour lengths predicted for a linear repressor–operator complexes (Figure 1C, green squares). The clustering of the points at this particular position shows that many of the 205 complexes have repressor specifically bound at the operator sequence, rather than associated nonspecifically with the DNA or surface. The remainder of the complexes with the sum of arm lengths equal to 169 ± 16 nm have a more uniformly distributed combination of arm lengths, as would be expected for complexes in which the repressor is bound to the linear DNA in a sequence-independent manner.

In addition to these linear repressor–DNA complexes, we observed another cluster (Figure 1C, red square) of complexes with approximately equal arm lengths but shorter total DNA length (∼120 nm). Both the individual arm lengths and their sum agree with those expected for specific looped complexes in which a repressor binds simultaneously to both operator sites (see Materials and Methods). These results demonstrate that Lac repressor can form looped complexes on both in-phase and out-of-phase di-operator molecules, even with small loop sizes. Single-operator DNA bound to Lac repressor is not appreciably bent [20]. Consistent with the expected structures, we found the angles at which the DNA arms exit from the protein to be significantly more acute for the looped than the unlooped complexes (see Materials and Methods).

### DNA Wraps around the Repressor in Looped Complexes

Although Lac repressor–DNA looped complexes with short operator spacings have been examined by electron microscopy [15], they have not been imaged with sufficient resolution to determine the position of the DNA relative to the protein in the three-dimensional structure of the complexes. Two kinds of models have been proposed: one in which the bulk of the repressor is positioned external to the looped segment of DNA (Figure 1D) (for example, [26]) and one in which the repressor is positioned in the center of the DNA loop, possibly making stabilizing contacts with the looped segment (Figure 1E) (for example, [24]).

To determine whether the AFM images are capable of differentiating between these two types of proposed structures, we modeled repressor and DNA as simple geometrical solids (with overall dimensions corresponding to those determined crystallographically) positioned flat against a surface, with the repressor within (Figure 1E) or outside of (Figure 1D) the DNA loop. The corresponding AFM images were then computed using a numerical simulation that accounts for the image distortion caused by the shape and finite width of the AFM tip [57]. The simulated images clearly show that these two conformations should be differentiated by AFM. Although the images of looped repressor–DNA complexes have a variety of shapes (Figure 1F), we do not observe any complexes (out of 102 analyzed) that are consistent with the simulated image (Figure 1D) in which the repressor protein lies outside of the DNA loop. In contrast, many images similar to that simulated for the repressor lying within the loop are seen (e.g., Figure 1F). Assuming that at least some complexes adhere to the surface in the orientation modeled, the AFM images exclude the structure of Figure 1D and strongly favor a model in which the repressor is positioned within the DNA loop.

### Two Looped Species That Can Directly Interconvert Are Formed with an In-Phase Di-Operator DNA

**Figure 2:**
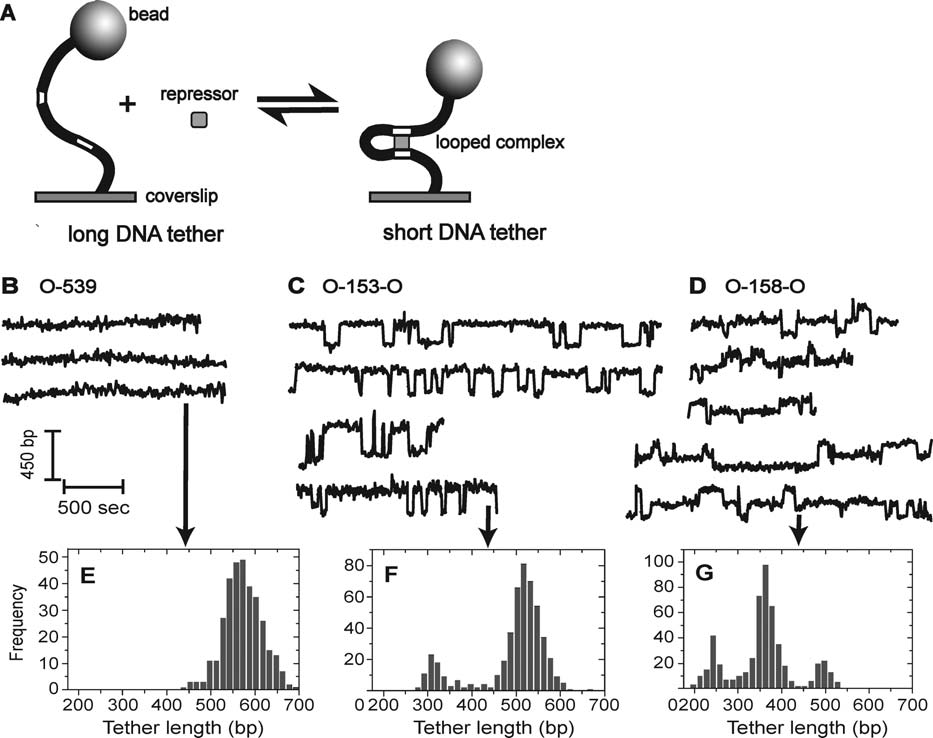
Dynamic Repressor-Mediated Loop Formation in Single DNA Molecules at Equilibrium (A) Experimental design (not to scale). Left: a digoxigenin- and biotin-labeled DNA (black curve) containing two operator sites (white bars) is immobilized at low surface density on an anti-digoxigenin–coated coverslip; the distal end of the DNA is labeled with an avidin-conjugated 0.098-μm diameter polystyrene bead for visualization by light microscopy. Right: formation of a Lac repressor-mediated looped complex decreases the effective length of the DNA tether and thus reduces the observed Brownian motion of the tethered bead. (B–D) Examples of Brownian motion recordings for single beads tethered by either O-539 (B), O-153-O (C), or O-158-O (D), in the presence of 5.4 nM Lac repressor. The extent of Brownian motion is expressed as the effective tether length, the length of DNA that gives the same amount of Brownian motion in the absence of repressor (see Materials and Methods). (E–G) Effective tether length histograms (bin width 15 bp) from the individual records designated by arrows.

To characterize the dynamics of Lac repressor–induced DNA looping, we used TPM to monitor changes in effective DNA length in real time. Before examining Lac repressor–induced DNA looping with constructs containing two operator sites (Figure 2A), we performed control experiments using two DNA fragments, each containing a single operator site (O-539 and O-349; Figure 1A). In the presence of repressor, both DNAs exhibited effective lengths that are indistinguishable from the tether lengths of the DNA in the absence of repressor (Figure 2B and 2E, and unpublished data). Thus, repressor binding to a single operator site or to nonspecific sites does not change the effective length of the DNA tether. This observation confirms that Lac repressor does not induce appreciable bending in single-operator DNA [45,58].

In contrast, for beads tethered by two-operator O-153-O DNA in the presence of Lac repressor (Figure 2A), time records of bead Brownian motion (expressed as effective DNA tether length; Figure 2C) alternate stochastically between a long-tether-length (unlooped) state and a short-tether-length (looped) state. This parallels previous observations using di-operator DNAs with larger interoperator separations [23,42,43,59]. Tether length distribution histograms from individual data records (e.g., Figure 2F) typically have two peaks. In the example shown, the peak centered at approximately 525 bp corresponds to unlooped O-153-O (length 539 bp) and a second peak at approximately 310 bp corresponds to the looped DNA. Measured TPM peak positions can vary slightly from molecule to molecule because of small differences in positioning of the molecules with respect to the microscope focal plane [41]; it is therefore more precise to measure peak spacings than to measure absolute positions. In a set of 16 records, the mean spacing between the two histogram peaks was 204 ± 37 (S.D.) bp. The observations of two TPM peaks with a well-defined spacing further support the AFM data demonstrating that Lac repressor can form specific looped complexes even when the operators are out of phase.

Unexpectedly, when the spacing between the operators was increased by 5 bp to 158 bp, three (not two) discrete tether lengths are observed in the presence of repressor (Figure 2D) in 49 out of 70 records. In the other 21 records (most of which are of comparatively short duration), only two of the three histogram peaks were clearly discernable. In the example shown, the peaks are at approximately 500, 360, and 250 bp (Figure 2D and 2G). The observed tether lengths of approximately 500 bp are assumed to arise from the nonlooped states of the 544-bp-long DNA. Consistent with this assumption, only tether lengths of approximately 500 bp were observed with both the O-153-O and O-158-O constructs in control experiments in which 0.2 mM isopropyl β-D-thiogalactoside (IPTG) was added to block repressor–operator binding or in which repressor was omitted (unpublished data). In the 36 tether-length records in which the positions of the three peaks could be most reliably measured, the positions of two shorter tether-length peaks were 114 ± 29 (S.D.) bp and 228 ± 40 (S.D.) bp less than that of the approximately 500-bp unlooped peak, with the 114-bp shorter peak (∼386 bp) being the predominant species. These results indicate that Lac repressor–di-operator complexes can exist in two different looped conformations when bound to O-158-O. If the two structures interconvert directly, they likely involve two distinct conformations of the repressor. In contrast, if the two species can interconvert only by going through the unlooped state, there is the additional possibility that the two species are topological isomers that differ in the configuration of the DNA loop but in which repressor conformation does not change significantly [19,26,28,60]. Interconversion of such topoisomers requires transient dissociation of one of the two operator sites from the repressor.

To distinguish between these possibilities, we further analyzed TPM records of O-158-O (such as those in Figure 2D) and counted the relative number of times the short-tether loop state was immediately followed by the long-tether loop state rather than the unlooped state. This partition ratio, *P*′′_short-tether→long-tether/short-tether→unlooped_ = 3.0 ± 1.1 (see Materials and Methods) is significantly greater than one, demonstrating that conversion to the long-tether loop is the favored pathway of exit from the short-tether loop. Similarly, the long-tether loop preferentially converts to the short-tether loop relative to converting to the unlooped species, with *P*′′_long-tether→short-tether/long-tether→unlooped_ = 2.3 ± 0.5. These observations show that the two looped states can interconvert directly without passing through the unlooped state. Thus, they contain distinct conformations of the protein; they are not simply topological isomers that differ (for example) in operator binding orientation [28,60].

The single looped species seen with O-153-O has a tether length significantly different (*p* < 0.025; *t*-test with 50 d.f.) from those of either O-158-O looped species. Since the lengths of the two DNAs differ by an amount (5 bp) too small to detect by TPM, the observed differences in the tether lengths of the three distinct looped species are likely due to differences in the preferred angle between the DNA arms as they exit the loop. This in turn strongly suggests that the looped segment of the DNA can have different three-dimensional geometries in Lac repressor looped complexes depending on operator phasing. Taken together, the O-153-O and O-158-O data indicate that Lac repressor can induce substantially different loop structures depending on operator spacing and that a single di-operator-repressor complex can exist in multiple (at least two) different looped conformations.

### In- and Out-of-Phase Loops Have Similar Stabilities

In crystal structures of intact tetrameric Lac repressor, the two DNA binding domains have roughly similar orientations [20]. They are therefore well situated to form looped complexes with DNAs such as O-158-O, in which the two operator sites are separated by an integral number of helical turns putting them on the same face of the double helix. In contrast, the operator sites are positioned on opposite faces of the DNA in O-153-O. If looped complexes formed by O-158-O and O-153-O are identical in three-dimensional structure except that the DNA has no torsional strain in the former and one-half twist between the operators in the latter, the equilibrium constant for loop formation with O-153-O should be approximately 10^4^-fold lower (see Materials and Methods) than that for O-158-O due to the significant energetic cost predicted by standard models of DNA mechanics of introducing a half twist into the 153-bp DNA segment.

The TPM looping experiments permit thermodynamically rigorous measurement of the equilibrium constant for loop formation as the ratio of the total time spent in a particular looped state to the total time spent in the unlooped state. Under the conditions of the experiment, these equilibrium constants are 0.5 ± 0.1, 2.9 ± 0.5, and 0.39 ± 0.08 for the O-153-O loop, O-158-O long-tether loop, and O-158-O short-tether loop, respectively. The long-tether loop is approximately 7-fold more populated than short-tether loop and therefore is the preferred looped conformation for O-158-O. Although the O-153-O looped complex is less stable (relative to the unlooped state) than the preferred looped complex of O-158-O, the equilibrium constants differ by only 5-fold, not the approximately 10^4^-fold expected from DNA twisting alone. This analysis suggests that the O-153-O and O-158-O looped complexes may incorporate significantly different repressor conformations that accommodate the different operator spacing without significantly twisting the interoperator DNA.

### Kinetic Mechanism of Looping and Loop Interconversion

**Figure 3:**
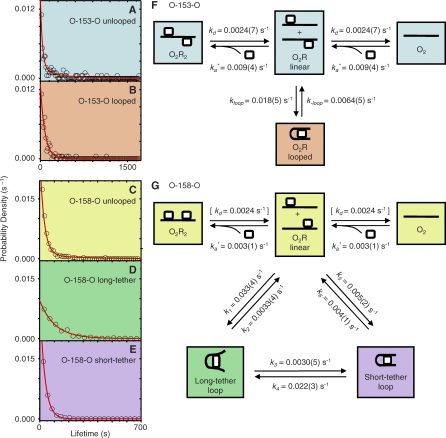
State Lifetime Distributions (A–E) and the Mechanisms and Rate Constants Determined from the TPM Data (F and G) For each state, *N* measured lifetimes were used to construct a histogram (circles) of bin width *W*, which is plotted here as a scaled probability density (see Materials and Methods); also shown (lines) are the theoretical distributions calculated from the mechanisms and rate constants. (A) O-153-O unlooped state (*N* = 110, *W* = 15 s); (B) O-153-O looped state (*N* = 108, *W* = 18 s); (C) O-158-O unlooped state (*N* = 168, *W* = 15 s); (D) O-158-O long-tether loop (*N* = 245, *W* = 40 s); (E) O-158-O short-tether loop (*N* = 80, *W* = 30 s). (F and G) Proposed mechanisms for repressor-mediated looping of O-153-O (F) and O-158-O (G). O_2_ (lines), di-operator DNA molecule; R (squares), repressor tetramer. For simplicity, only one of the two identical reaction steps linking each of the two equivalent O_2_R linear species with the other states is shown; the rate constants are the microscopic rate constants for each of the individual reaction steps. Similarly, unlooping rate constants *k*_-loop_, *k*_2_, and *k*_6_ are for the dissociation of repressor–single operator interactions; there are two such interactions in a loop so that the overall unlooping rate constant is twice the value given. k_a_* is the pseudo first-order rate constant for repressor association with operator. Numbers in parentheses are the standard error (S.E.) of the final digit of the corresponding rate constant. Rate constants were determined from observed state lifetimes, equilibrium constants, and kinetic partition ratios as described in Materials and Methods. The O-158-O rate constant in brackets was not well determined by the experimental data and was instead assumed to be equal to the corresponding O-153-O rate constant (see Materials and Methods).

To more fully characterize the mechanism by which repressor interacts with O-153-O, the complete set of O-153-O TPM records (e.g., Figure 2C) was analyzed to determine the lifetime distributions of the unlooped (Figure 3A) and looped (Figure 3B) states. The loop lifetime histogram is well fit by a simple exponential function, consistent with the interpretation that the O-153-O looped state is a single chemical species, not an unresolved mixture of two states. In contrast, the unlooped state lifetime histogram requires a distribution function that is the sum of at least two exponential terms to produce an acceptable fit. A multiexponential distribution is expected [23] because we know a priori that an unlooped di-operator DNA can exist in a minimum of four different states, an “O_2_R_2_” state that has two bound repressor molecules, two equivalent “O_2_R linear” states in which a single repressor molecule interacts with one operator, and an “O_2_” state with no bound repressor. These four unlooped species, together with the single looped species, comprise the minimal kinetic scheme (Figure 3F) for the interaction of repressor with O-153-O DNA. The scheme has only four independent rate constants; because the TPM experiments provide the shapes of the lifetime distributions (for both the looped and aggregate unlooped states) and the equilibrium constant (between the looped and aggregate unlooped states), they allow determination of well-constrained values for all four (Figure 3F; see Materials and Methods). The shapes of the lifetime distributions predicted by this scheme reproduce the empirical data within experimental uncertainty (Figure 3A and 3B); similarly, the value of the equilibrium constant predicted by this scheme and that determined by experiment are also in good agreement (0.48 and 0.53 ± 0.11, respectively).

In the analysis of O-158-O state lifetimes, both the long-tether and short-tether looped state distributions were fit well by single exponential functions (Figure 3D and 3E), consistent with mechanisms in which each looped state consists of a single chemical species. O-158-O is expected to have the same four unlooped states as O-153-O, leading to the kinetic scheme of Figure 3G. In addition to the three lifetime distributions, the TPM experiments also directly measure the two equilibrium constants and three partition ratios. These data taken together with the value of the repressor–operator dissociation rate constant, *k*_d_, measured in the O-153-O experiment permitted precise determination (see Materials and Methods) of the forward and reverse rate constants for reaction pathways in the kinetic scheme (Figure 3G). As with O-153-O, the kinetic scheme with the deduced rate constants reproduces the lifetime distributions (Figure 3C–3E) and other experimental data. The values calculated for *k*_3_ and *k*_4_ are significantly greater than zero, confirming that all features of the TPM data (not merely the partition ratios) are consistent with the ability of long- and short-tether loops to interconvert directly without going through the O_2_R linear species. In contrast, when we performed a separate kinetic analysis using the scheme of Figure 3G but with fixed *k*_3_ = *k*_4_ = 0 (unpublished), the mechanism predicted values of equilibrium constants and partition ratios substantially different from those measured, confirming that direct interconversion of the two looped states is necessary to explain the experimental observations through the mechanism of Figure 3G. Finally, the rates of interconversion are consistent with observation that the two species are not resolved in gel mobility shift experiments (unpublished data; see also [15]), because they interconvert on a time scale (<1 min; Figure 3G) that is much shorter than the time required for electrophoresis.

## Discussion

We have demonstrated that Lac repressor can form stable looped structures containing short loops even when the operators are out of phase. In addition, a single looped complex can exist in an equilibrium of two different conformations. Although the TPM experiments indicate that the preferred conformations of looped complexes are different for the in-phase and out-of-phase DNAs, the AFM data show that all complexes have the DNA wrapped around Lac repressor. Taken together, our data strongly suggest that Lac repressor undergoes large-scale conformational changes that can stabilize loop structures by reducing the DNA strain required for loop closure. In the sections that follow, we discuss the observations in the context of possible loop structures and protein conformational changes.

### A Large-Scale Change in Lac Repressor Three-Dimensional Structure

As previously discussed, a likely cause of the existence of two loop structures that are in equilibrium with one another for the in-phase O-158-O DNA is a conformational change in the repressor protein itself. However, the effective tether lengths of the two looped species differ by 114 bp, corresponding to a DNA contour length difference of 38 nm. As this distance is larger than the overall dimension of the repressor protein itself, only a reconfiguration of the repressor structure that substantially changes the angle between the two DNA arms as they exit from the loop is likely to account for the detected difference between the two types of loops. We previously showed that a single acute DNA bend could shorten the observed TPM tether length by 284 bp [45]. Thus, a large-scale repressor conformational change that changes the relative orientation of the operator-binding domains could be sufficient to explain the difference in tether length measured for the two O-158-O loops.

**Figure 4:**
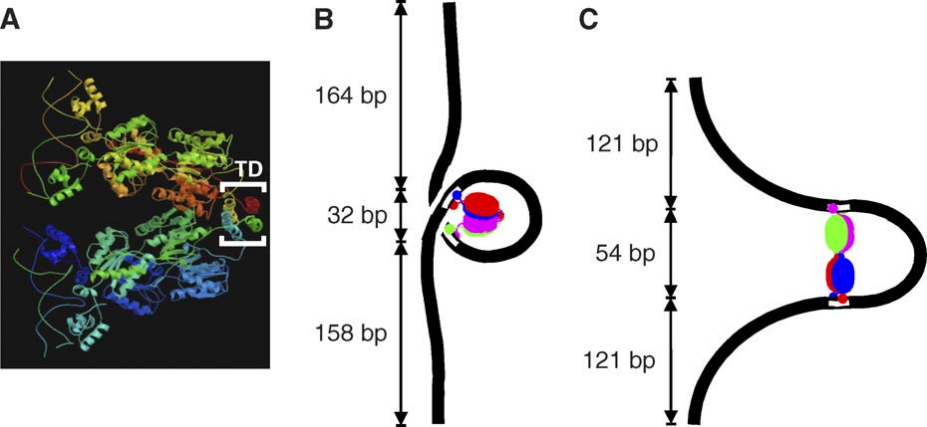
Lac Repressor and Proposed O-158-O Loop Geometries (A) Crystal structure of Lac repressor complexed with two 21-bp symmetric operator DNAs (Protein Data Bank 1LBG; [20]). Each N-terminal headpiece of the tetrameric repressor is in contact with DNA (shown as thin wires). The C-terminal α-helical coiled-coil domains from each of the four subunits associate to form the stable 4-helix bundle tetramerization domain (TD). The center-to-center spacing of the two DNAs is approximately 7 nm. (B and C) Proposed models and predicted effective tether lengths for long-tether (B) and short-tether (C) loops. Black curves and white bars represent flanking DNA and operator sites, respectively. Each of the four subunits of the repressor is shown in a different color. Structures are shown as if flat (for clarity), but actual loop structures are at least somewhat nonplanar. (B) Long-tether loop. The repressor has the crystallographic V-shaped conformation. (C) Short-tether loop. The repressor has opened up to a fully extended form by rotating the two dimers about the axis of the 4-helix bundle, which is roughly normal to the plane of the figure.

Prior studies raised the possibility that Lac repressor may in fact be capable of such a large-scale conformational change. Crystal structures of intact tetrameric Lac repressor [20], either alone or in complex with operator DNA, show an asymmetrical “V” structure with two operator-binding domains located at the tips of the arms of the V (Figure 4A). The major contacts between the two halves of the tetramer are restricted to the 4-helix bundle at the tip of the V. Friedman et al. [19] and Lewis et al. [20] both noted that spreading open of the V into a more extended conformation would result in only a small increase (∼300 Å^2^ [20]) in water-accessible surface area and thus is predicted to be only moderately unfavorable in free energy. Indeed, Friedman et al. [19] speculated that such an extended conformation might facilitate looping of DNA with closely spaced operators. Early solution X-ray scattering, powder diffraction, and electron microscopy results are consistent with an extended conformation [61,62]. A more recent electron microscopy study suggested that both a compact and an extended conformation of repressor may coexist in solution [63]. Finally, hyperstable looped complexes formed from di-operator DNAs that contain static bends between the operators may have at least two alternative loop shapes, depending on the phasing of the bend and operators [26,35]. Fluorescence resonance energy transfer measurements are consistent with a “V” repressor structure in one of these loops, and a more extended structure has been proposed for the other [35,36]. Our results lend further credence to the idea that Lac repressor can exist in a V-shaped and an extended conformation and in addition show that the two structures can exist in dynamic equilibrium in the same protein–DNA complex.

### Three-Dimensional Structures of the Two In-Phase Loops

**Figure 5:**
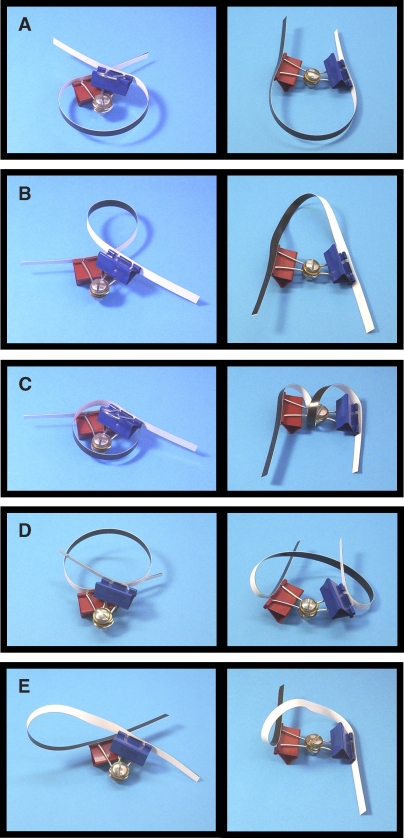
Physical Models Corresponding to Possible In-Phase Loop Geometries Photographs show loop models with the V-shaped repressor conformation (left column), and the corresponding configurations that result when the repressor V is opened (right column). In each pair, the structure on the right was produced from that on the left by rotating the two half-tetramers (represented by a blue or red paper clip) away from each other about the axis of the 4-helix bundle (represented by a silver bolt); the front dimer is rotated approximately 80° clockwise and the rear approximately 80° counterclockwise. (A) Structures proposed (Figure 4) for the O-158-O long- (left) and short-tether loops (right). Left structure is similar to the “wrapping toward” model of Friedman et. al. [19], but with the DNA helix axes roughly perpendicular to the 4-helix bundle axis, as seen in the repressor–oligonucleotide co-crystal [20]. (B) “Wrapping away” loop (left) [19,35]. (C) Alternative wrapping toward loop [20,24,35]. (D) Alternative wrapping away loop. (E) Simple loop [19,20]. The right-column structures (B) through (E) are predicted to be significantly less stable than that in (A) due to increased twisting strain (B), bending strain (C and D), or both (E). The paper strip representing the DNA is colored black on one side and white on the other to make any twist visible. The structures (A) through (E) are topologically equivalent to the P2, P1, P1, P2, and A2 configurations described by Swigon et al [28]. Loops with additional antiparallel configurations (A1 and A1*; not shown) are also possible, but all such antiparallel configurations produce highly strained opened structures similar to that shown for (E). In classifying the structures, we assume that the flanking DNA arms are constrained, either by attachment to surface and bead (as in the experiments reported here) or by incorporation into a larger DNA circle (as in a plasmid or E. coli chromosome). With constrained flanking DNA arms, the two P2 structures (A and D) are topologically distinct; they can only be interconverted by temporarily separating a DNA binding domain from its operator or by passing the loop segment through one of the arms. The same is true for the two P1 structures (B and C).

To assess the possible structures that could explain our TPM and AFM data, we made a comprehensive delineation (Figure 5, left column) of the types of loop geometries that can be made with the repressor in its crystallographic conformation and a small interoperator spacing DNA, together with the loop configurations predicted to arise from these structures if the repressor is distorted into an extended conformation by pivoting the two core dimers about the axis of the 4-helix bundle (Figure 4A). The analysis assumes a simple rigid-body mechanical model in which this pivoting is the only permitted structural change in the repressor. As noted previously, we consider “wrap away” models such as that of Figure 1D (Figure 5B and 5D, right) to be unlikely because they are inconsistent with the colocalization of DNA loop and repressor protein we observe by AFM. More significantly, the left-column structures of Figure 5B–5E produce strained, energetically implausible configurations if the repressor is opened to the extended conformation without allowing one of the DNA-binding domains to temporarily dissociate from the operator to relieve the strain. Thus, the structures in Figure 5A are the only loop geometries that adequately explain the observed ability of the long- and short-tether states to interconvert directly without passing through an unlooped configuration. Although the loop structures in Figure 5B–5D have been previously proposed, they were proposed for DNAs that differed materially from the ones used here because the DNAs had permanent bends [35], were bent by the catabolite activator protein [20], or had a considerably shorter interoperator spacing [20,24,35]. As previously noted [28,35,64], it is quite possible that the lowest energy loop structure would be different in such different circumstances. Loop configurations other than those shown in Figure 5 are possible if the repressor is capable of other structural changes (e.g., headpiece reorientation; see [1,28]) in addition to the simple hinge motion considered here; however, there is little experimental evidence for such changes. Steered molecular dynamics calculations [1] that allowed the operator axis to rotate to a position nearly perpendicular to the plane of the Figure 4A illustration produced wrap-away loop structures that appear inconsistent with our AFM data. However, these calculations used a smaller interoperator spacing which could direct formation of different loop geometries than those obtained with the approximately 150-bp spacings used here.

To determine whether the two loop structures in Figure 5A can account for the two tether lengths observed with O-158-O, we made more detailed models based on crystallographic and electron microscopy data [19,20,63]. In one model (Figure 4B), the repressor adopts the V-shaped conformation determined by X-ray crystallography and the DNA loop wraps around the repressor in the fashion analogous to the “wrapping toward” loop model of Friedman et al. [19]. The interoperator segment has near-zero twist. In the other model (Figure 4C), we propose that the repressor is in an almost fully extended conformation with a shape similar to that inferred from electron microscopy [63]. In this model, the repressor is still positioned in the interior of the curved DNA segment and the interoperator segment is still not twisted. A rough calculation (see Materials and Methods) of the effective tether lengths predicted by these loop models yields 354 and 296 bp for the V-shaped and extended repressor conformation, respectively. Given the approximate nature of the calculation, these lengths are in reasonable agreement with the values (421 ± 143 [S.D.] and 309 ± 132 bp) measured by TPM and suggest that the long-tether-length conformation is the one in which the Lac repressor is in the V-shaped conformation (Figure 4B) seen in the crystal structures [19,20].

**Figure 6:**
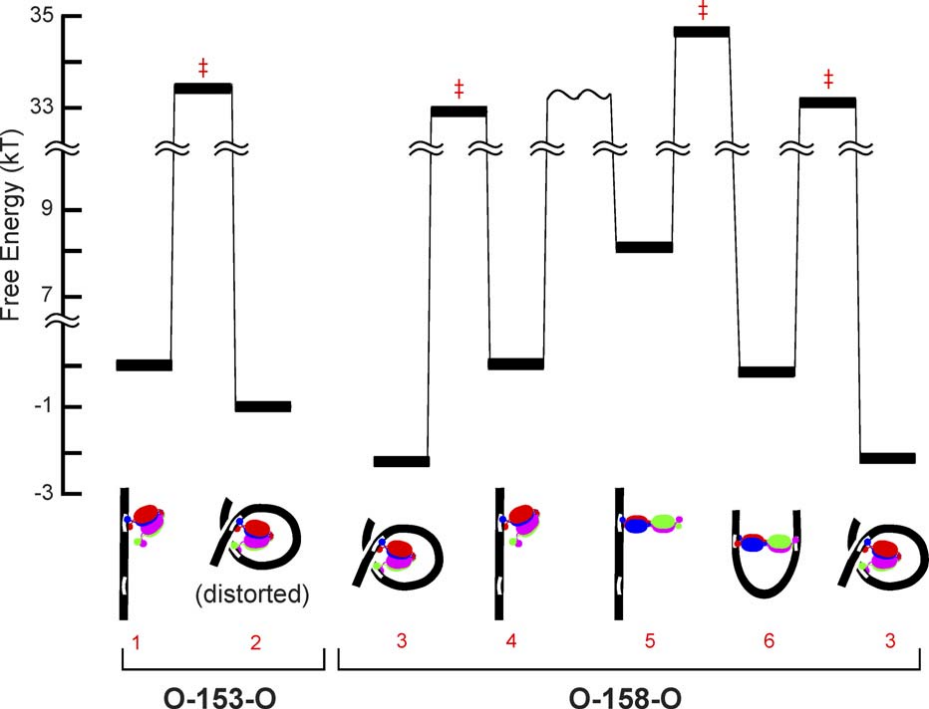
Free-Energy Profile for the Looping and Unlooping Reactions Free energies (*G*) of the stable species, and inferred *G* of transition states (‡) for looping/unlooping reactions, are shown with cartoon representations of the three-dimensional structures proposed (see Figure 4B and 4C, and text) for each stable species. The linear complexes with the repressor in the V” conformations (species 1 and 4) were chosen as reference states and assigned *G* = 0; the relative energies of other species were calculated from the rate constants (Figure 3). Transitions state energies are rough estimates based on the assumptions that all reactions are single-barrier processes with identical pre-exponential factors arbitrarily set at 6.1 × 10^12^ s^−1^. The conformational change of the repressor bound to linear DNA (species 4 ↔ 5) cannot be detected in the experiments; *G* of species 5 is instead estimated from the change solvent-exposed protein surface area (see Materials and Methods), and the transition state is not shown. The three-dimensional structure of species 2 is assumed to be similar to that of species 3, except that the repressor structure is distorted so as to minimize DNA twist (see text). Species 3 is shown twice so that all transitions in the cyclic O-158-O looping scheme (Figure 3G) can be shown. Free energies are expressed as multiples of the product of the Boltzmann constant (*k*) and the absolute temperature ( *T* ).

The measured difference in free energy between the two in-phase loops is small, amounting to little more than twice the energy of thermal agitation (Figure 6, species 3 and 6). The small energy difference is consistent with the energies estimated for the proposed loop structures (see Materials and Methods), demonstrating that the proposed structures are good models for the two looped species seen with O-158-O. The closure of the loop is predicted to be energetically downhill for both structures (Figure 6, species 4 → 3 and species 5 → 6) because the formation of the highly favorable repressor–operator interaction more than compensates for the energy required to bend the DNA. Although opening the repressor “V” structure (species 4 → 5) is expected to be energetically costly because of the elimination of some favorable subunit–subunit interactions, this energy cost is almost fully offset by the fact that the DNA bending strain required to form the extended repressor loop structure (species 5 → 6) is less than that required for the “V” repressor loop structure (species 4 → 3). This difference in DNA bending strain also explains the measured kinetics of loop closure. In the transition state for a loop closure (Figure 6, species 4 → 3 or species 5 → 6), the DNA is bent into a shape very close to that of the fully formed loop and the favorable repressor–operator contacts are just beginning to form. The energy input required to surmount the transition state barrier in going from species 5 → 6 is lower than that in going from 4 → 3; this is readily explained by the smaller DNA bending required for the former. In summary, the proposed conformations in Figure 4B and 4C are consistent with both the kinetics and thermodynamics measured for formation of the two kinds of in-phase loops. In addition, conformations appearing consistent with both of the proposed structures can be seen in the AFM images (Figure 1F). Nevertheless, our structural conclusions are based on a simple rigid-body model of the Lac repressor protein in which pivoting around the four helix bundle is the only allowed internal motion. The conclusions may require revision if future experiments reveal that additional regions of the repressor structure can hinge or deform during loop formation.

### Conformational Flexibility of Lac Repressor Can Account for the Stability of Out-of-Phase Loops

The WLC model of DNA mechanics predicts a substantial energy cost to twist an approximately 150-bp segment of DNA by a half turn. Although this cost might be reduced substantially by local denaturation or kinking of the DNA, the rate at which such discontinuities form spontaneously in DNA is likely too low to explain the rates of loop formation observed here [30–33]. Based on the WLC model, the out-of-phase looped structure would be expected to be greatly destabilized relative to the in-phase structure if the repressor protein has the same conformation in both cases. Our data show that even with an out-of-phase interoperator spacing as small as 153 bp, Lac repressor can form highly stable loops. The rough features of these out-of-phase looped complexes are remarkably similar to those of the two different O-158-O in-phase loops: the free energies are similar (Figure 6, species 2, 3, and 6); the tether length of the O-153-O loop is between those measured for the O-158-O loops; and all loops appear in AFM images to have the same wrap-around configuration with only subtle differences in the observed DNA exit angles (Figure S1) that might be attributed to differences in conformation of the looped species. Most importantly, the energy of the transition state for closure of the out-of-phase loop (Figure 6, species 1 → 2) is almost identical to that for the closure of the long-tether in-phase loop (Figure 6 species 4 → 3), even though the strain in the out-of-phase DNA construct would be expected a priori to be the same or larger. If formation of the out-of-phase loop requires the prior formation of the energetically unfavorable extended form of the repressor protein, which is not necessary for the formation of the long-tether in-phase loop (Figure 6, species 3) but is necessary for the short-tether in-phase loop (Figure 6, species 6), the loop closure transition state energy for 1 → 2 would be expected to be higher than that for 4 → 5 → 6, which is inconsistent with the kinetics we measure (Figures 3 and 6). Therefore, the simplest hypothesis is that the out-of-phase loop has the same repressor configuration as the long-tether in-phase loop, for which we propose a structure with the “V” repressor conformation. Nevertheless, the detailed three-dimensional structures of species 2 and 3 must be different to accommodate the different operator phasing; this difference is also necessary to account for the somewhat different tether lengths and gel mobility shifts [15] observed for 153 and 158 loops. Species 2 is unlikely to contain a highly strained structure of the repressor because its free energy is similar to that of species 3. Instead, we propose that closure of the out-of-phase loop captures a dynamic conformation of an already flexible region of the repressor. One possible hypothesis is that the DNA binding headpieces move apart (by rotation away from the plane of the illustration in Figure 4A), thereby reducing or eliminating the twist in the loop. Other modes of conformational flexibility in the repressor [1,28] could also account for the stability of the out-of-phase loop.

Although we observe only a single tether length for the out-of-phase loop (and only two tether lengths of in-phase loops), it is clearly possible that each DNA can form additional loop structures (possibly including those in Figure 5B–5E) that are not detected in our experiments because they are not sufficiently thermodynamically or kinetically stable to be seen.

### Alternative Loop Structures and *lac* Operon Regulation

The idea that two or more different looped species can form on DNAs with small operator spacings is consistent with previous observations that 153- and 158-bp spaced operators produce putatively looped species with substantially different gel mobilities [15]. Swigon et al. [28] showed that various alternative loop structures are energetically accessible and also demonstrated that earlier DNase I footprinting data on loops with small interoperator spacings [15] are consistent with a structural model incorporating an extended-repressor loop. Kahn and coworkers [26,35,36] used a variety of techniques to study the hyperstable loops formed from DNAs with strong intrinsic bends in the interoperator segment. These studies demonstrate that the mechanical constraints imposed by bends at different positions relative to operators induce formation of alternative loop structures. However, they do not determine whether the alternative structures have substantially different repressor conformations or are merely loop topoisomers. While this manuscript was under review, Normanno et al. [59] reported that twisted DNA with much larger operator spacings can form two alternative types of loops. The three-dimensional structures of these loops and the extent to which they correspond to the alternative structures of small loops that we observe is unclear, particularly since multiple topoisomeric species have similar energies in the limit of large operator spacing [28]. Our studies demonstrate two looped states in DNA without static bends and further show that these must arise from a large-scale structural reorganization of the repressor itself. Furthermore, the experiments directly demonstrate that different kinds of loops can form at equilibrium from the same DNA. By going beyond analysis of equilibrium structure to examine the dynamics of looped species formation, breakdown, and interconversion, we also show that these processes occur on a timescale (tens to hundreds of seconds) similar to that of rapid transcriptional responses to changes in environmental conditions. The dynamic data also provide key clues about loop configuration that lead to our proposal of novel loop geometries with decreased DNA bending or twisting strain energies relative to previously proposed structures. Recent studies support the notion that alternative loop structures like those proposed here are necessary to quantitatively account for the extent of looping-mediated repression in measured in living cells [38,39].

On the basis of DNA mechanics alone, the DNA loops formed by Lac repressor in vivo would be expected to have enormous differences in stability. Loops between operator pairs separated by distances both much larger than and much smaller than the persistence length of DNA (and hence, with predicted DNA bending energies both much larger and much smaller than the thermal energy ½*RT*) are important for repression. Also, changes in the extent of supercoiling, the binding of DNA bending proteins (e.g., catabolite activator protein), and association with proteins that alter DNA flexibility [17,43,65] could cause dramatic differences in the mechanics of loop closure and thus greatly perturb loop stability. The ability of Lac repressor to dynamically alter its conformation (and thus, the geometries of and mechanical strain in the resulting loops) that is observed here suggests the repressor may have evolved the ability to produce polymorphic loop structures as a way of stably maintaining regulation of transcription under conditions of widely varying mechanical difficulty of loop formation. This is a testable hypothesis—it predicts that mutant repressor proteins designed to have decreased conformational flexibility should be less able to achieve stable repression both in vivo and in vitro over a range of conditions thought to alter DNA mechanics.

## Materials and Methods

### Materials.

Lac repressor was a kind gift of Kathleen Matthews (Rice University). All repressor concentrations are expressed as the concentration of tetramer. Avidin-conjugated beads (0.098-μm diameter) were prepared as described [23].

### Plasmids.

Di-operator and mono-operator parent plasmids pH108 (contains two symmetric operators separated by 114 bp) and pH109 (identical to pH108 except that three point mutations in one of the operator sites abolish recognition of that site by repressor) were gifts of Sankar Adyha [66]. Two-operator plasmids pOKW153 and pOKW158 were constructed from pH108 by inserting 39-bp (5′-GTTACCTTAGGTACCACTAG-TCTAGAATGCATTCCGCGG-3′) or 44-bp (5′-GTTACCTTAGGTACCACTAG-TCTAGACCGCGGAGATCTCAATTG-3′) linkers, respectively, into the unique BstEII site. One-operator plasmid pOKW153C, which is identical to pOKW153 except for the three point mutations, was constructed by inserting the 39-bp linker into the BstEII site of pH109.

### Oligonucleotide derivatives and operator DNAs.

5′ Digoxigenin-labeled oligonucleotide P31-dig (digoxigenin-5′-TCGATAGCG-TGATCGTGC-3′) and 5′ biotin-labeled oligonucleotides P32-bio (biotin-5′-CGTATCA-CGAGGCCCTTT-3′) and P86-bio (biotin-5′-CAATAATTTATTCCATGTCAC-3′) were synthesized from the corresponding 5′ amino-labeled oligonucleotides by reacting with either digoxigenin-3-O-methylcarbonyl-ɛ-aminocaproic acid-NHS ester (Roche Applied Sciences) or biotin-XX-NHS ester (Glen Research). Labeled oligonucleotides were subsequently purified by anion exchange high-performance liquid chromatography (HPLC). Mono- and di-operator DNA fragments O-153-O, O-158-O, and O-539 were generated by polymerase chain reaction (PCR) using primers P31-dig and P32-bio with pOKW153, pOKW158, and pOKW153C as templates. O-349 was made from pOKW158 using P31-dig and P86-bio. PCR products were purified by extraction with buffered phenol and with water-saturated 1-butanol, followed by four cycles of greater than 10-fold dilution and reconcentration in a Centricon-100 concentrator [67]. In constructs O-153-O and O-158-O, the center-to-center separation between the two operators are 153 bp (14.5 helical turns, assuming 10.5 bp/turn) and 158 bp (15 turns) respectively. Mono-operator construct O-539 is identical to O-153-O, except that only the biotin-proximal operator is functional in repressor binding (Figure 1A). In O-349, only the digoxigenin-proximal operator is present (Figure 1A).

### Tethered particle motion detection of DNA looping.

Formation and breakdown of repressor–DNA looped complexes formed from O-153-O or O-158-O were monitored using previously described single-molecule light microscopy techniques [23] with the following modifications: DNA–bead complexes were preformed by incubating 35 pM DNA with 0.56 nM avidin-conjugated beads in PTC buffer (20 mM Tris-acetate [pH 8.0], 130 mM KCl, 4 mM MgCl_2_, 0.1 mM EDTA, 0.1 mM DTT, 20 μg/ml acetylated BSA) for >60 min. Under this condition, the probability of having multiple DNA molecules attached to the same avidin-conjugated bead is less than 0.10. After attaching the DNA-bead complexes to the anti-digoxigenin–coated surface and washing the microscope flow cell with PTC buffer supplemented with 6 mg/ml casein, a solution of 5.4 nM repressor in LRB (10 mM Tris-HCl [pH 7.4], 200 mM KCl, 0.1 mM EDTA, 0.2 mM DTT, 5% DMSO, 0.6 mg/ml casein) was introduced. The cell was then monitored by video-enhanced differential interference contrast light microscopy at approximately 22 °C to observe the processes of looped complex formation and breakdown.

Time sequences of digitized images of DNA-tethered beads were collected using Glimpse (http://www.brandeis.edu/projects/gelleslab/glimpse/glimpse.html); each recorded image was the average of 64 consecutive video frames (2.1 s). Digitized images, together with the times at which each was acquired, were stored in the computer for subsequent off-line analysis. During the experiments, correct microscope focus was maintained automatically every 20 s by a stepper motor that moved the stage to the position giving the highest contrast in the image of a bead rigidly attached to the coverslip surface. Data acquisition was temporarily halted during the focusing process so that out-of-focus images were not included in the bead Brownian motion data.

The beads in TPM experiments experience a polymer confinement force directed away from the surface [68], but in these experiments, this force is expected to be insignificant because of the small (98 nm) bead diameter.

### TPM data analysis.

Brownian motion of DNA-tethered beads in each image was calculated as described [23], except that data were not filtered. Brownian motion records were converted to tether length measurements using a proportionality constant of 0.047 nm/bp obtained as described [41]. For O-153-O tethered beads, two Brownian motion states (looped and unlooped) were observed and their lifetimes were analyzed using the one-threshold discrimination algorithm [23]. Time records of O-158-O tethered bead Brownian motion with histograms that showed three discrete Brownian motion states were analyzed using an analogous two-threshold algorithm, with the thresholds positioned at the troughs between the histogram peaks representing the three states. The minority of records in which three states could not be clearly distinguished (see Results) were excluded from the analysis. Under the image acquisition conditions used for both O-153-O and O-158-O, states with lifetime < *t*_min_ = 10 s could not be reliably detected; therefore, such data were excluded from further analysis.

Measured lifetimes were binned and plotted as scaled lifetime probability density histograms


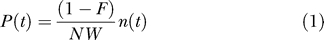


where *n*(*t*) is the number of events in the histogram bin centered at time *t*, *N* is the total number of observed events, *W* is the bin width, and *F* is the estimated fraction of missed events calculated as described below. Time constants for the theoretical lifetime distributions predicted by the kinetic schemes were calculated by the method of Colquhoun and Hawkes [69].

### State durations and missed events.

Under the image acquisition conditions used in the TPM experiments, occasions in which a DNA molecule existed in a particular looped or unlooped state for a time < *t*_min_ = 10 s could not be reliably detected. To estimate the number of such missed events, we first fit the raw lifetime histograms (using the Levenberg-Marquardt algorithm) for each looped state to the exponential distribution function [70]


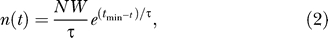


to obtain the time constant, *τ*, for each state. For the aggregated unlooped states of O-153-O and O-158-O, the corresponding bi-exponential function [71],


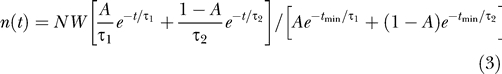


was instead used to obtain time constants *τ_1_* and *τ_2_* and amplitude *A* for each DNA. All fits were excellent with randomly distributed residuals.

For each looped state *i*, the fraction *F_i_* of events that were not detected (i.e., events with lifetime < *t*_min_) was calculated as


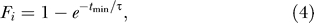


the mean lifetime *d_i_* was taken to be the lifetime distribution fit parameter τ, and its standard error was computed as


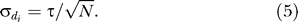


For the unlooped states, the corresponding equations were


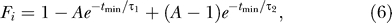



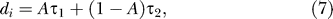


and


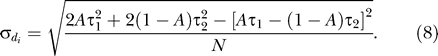


For any state, the total time *D_i_* in each state adjusted for the missed events, and its standard error σ*_Di_*, were calculated as


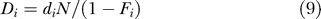


and


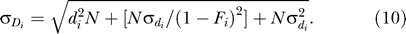


Results of these calculations are reported in Table S1.

### Equilibrium constants.

The equilibrium constant for loop formation, *K_i,j_*, is the ratio of the total time spent in state *i* to that spent in state *j*:


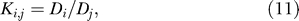


and the standard error of the equilibrium constant was calculated by error propagation as


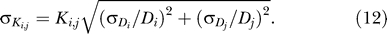


### Partition ratios.

To determine the partition ratios for the interconversion between the unlooped state and the two looped states of O-158-O, we first measured *C_a→b_*, the number of observed instances in the O-158-O TPM records in which state *a* was immediately followed by state *b*. This measurement was independently made for each pairwise combination of the unlooped, long-tether, and short-tether states. Only transitions in which both the beginning and ending states had lifetimes equal to or greater than 10 s were counted. Each *C_a→b_* was then used to calculate *C′_a→b_* and *C′′_a→b_*, values corrected for missed state *b* events and for both missed state *a* and state *b* events, respectively, as


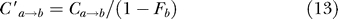



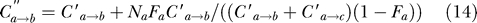


where *N_a_* is the number of state *a* events with lifetimes equal to or greater than 10 s and *F_a_* is the fraction of missed state *a* events as defined earlier. The partition ratio *P′′_a→b/a→c_* and its standard error *S′′_a→b/a→c_* were then calculated as


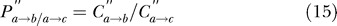



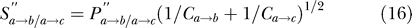


In all cases, the corrected partition ratios *P′′_a→b/a→c_* differ from the uncorrected values *P_a→b/a→c_* by less than 18%.

### Rate constants.

Molecules in the unlooped state can in principal interconvert between four different chemical species (O_2_R_2_, O_2_, and two equivalent O_2_R) before looping. The analytical expressions relating the O-153-O rate constants *k*_loop_, *k_d_*, and k_a_* (Figure 3F) to the unlooped state lifetime distribution are highly complex and therefore were not used. Instead, values and standard errors for the three rate constants (Figure 3F) were determined by numerical optimization to the observed set of unlooped state lifetimes using the MIL program [72,73] as implemented in the QuB software suite [74]. The analogous O-158-O rate constants (Figure 3G) were determined the same way, except that *k_d_* was held fixed at the value determined for O-153-O. That constraint was imposed because the values of *k_d_*, the single-operator repressor dissociation rate constant, are expected to be identical for the two DNA constructs because they have identical operator sequences. The apparent first-order rate constant for repressor–operator association, k_a_*, was allowed to vary in order to accommodate small unintended differences in the concentration of free repressor between the two experiments; however, the difference between the resulting values was roughly that expected merely from the calculated uncertainties. Preliminary fits of the O-158-O data in which both *k_d_* and k_a_* were allowed to vary did not adequately constrain the rate constant values because of the comparatively small difference between the two principal time constants for the O-158-O unlooped state lifetime distribution.

Rate constants related to looped state lifetimes (*k*_−loop_ in Figure 3F for O-153-O; *k*_1_ through *k*_6_ in Figure 3G for O-158-O) were determined by global optimization of the equations


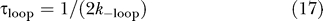



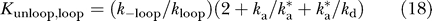


(for O-153-O), or


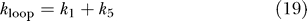



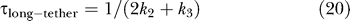



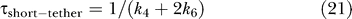



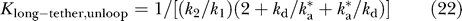



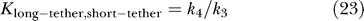



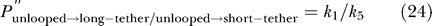



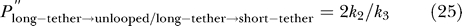



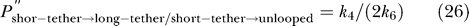


(for O-158-O) to minimize chi-square with respect to the values of the empirical quantities given on the left sides of the equations. Fits were weighted using the calculated standard errors of the empirical quantities; in cases in which the calculated error was less than 10%, a 10% error was assumed to allow for small systematic errors in the measurements. The calculated rate constants reproduced the experimental data almost exactly; all fit residuals were less than 15%. Error estimates for the rate constants were calculated by propagating errors from the empirical quantities using a Monte Carlo simulation [75]. All fitting and error propagation calculations were performed using custom computer software (available from the authors by request) implemented in MATLAB.

### AFM.

Di-operator DNA (40–60 nM O-158-O or 50 nM O-153-O) was incubated with 2-fold molar excess (over DNA) of repressor in LB buffer (35 mM Tris-HCl [pH 7.4], 140–180 mM KCl, and 0.3 mM EDTA) for 10 min at approximately 22 °C. An aliquot of the solution was then mixed with an equal volume of 1.7% glutaraldehyde in 10 mM Tris-HCl (pH 7.4) and incubated for 2 min at approximately 22 °C to allow protein–DNA cross-linking to occur. (This step helps to preserve the repressor–DNA looped complexes during the subsequent deposition process.) A volume of 1 μl of the cross-linked sample was then diluted with approximately 9 μl of DB buffer (10 mM Tris-HCl [pH 7.4], 10 mM MgCl_2_) and deposited onto a disk of freshly cleaved ruby mica (Asheville-Schoonmaker Mica Co.). After 1–2 min, the mica disk was rinsed with water and dried with a stream of nitrogen. AFM images were obtained in air with a Nanoscope IIIa microscope (Digital Instruments) operating in the tapping mode using high-frequency silicon tapping-mode cantilevers (f_0_ ∼ 330 kHz; Nanosensors). Images (512 × 512 pixels) were collected with a scan area of either 1.5 × 1.5 μm or 2 × 2 μm at a scan rate of two to four scan lines/second.

### AFM image analysis: DNA contour lengths and exit angles.

To distinguish Lac repressor–DNA looped complexes (in which protein is bound to both operator sites) from RO complexes (in which protein is bound to only one operator site) in the AFM images, we measured DNA arm length as the image contour length from the center of the protein to the end of each DNA arm using Nanoscope III (Digital Instruments) image analysis software. DNA contours were traced with short segments of straight line, and DNA arm length was obtained by adding the length of these line segments. The total image contour length of each complex was then calculated by summing the two DNA arm lengths. Measurements of the image contour lengths of 539- and 544-bp DNA molecules without bound repressor systematically underestimated the contour length by an average of 7.6% (14 nm); this underestimation is expected because the DNA path was approximated with line segments [55]. Since the DNA arms for both di-operator constructs are 190 or 196 bp (measured from the center of each of the operators to its closest 5′ end), the 7.6% systematic underestimation should lead to measured arm lengths of approximately 61 nm in the looped complexes. Therefore, all protein-bound molecules with both arm lengths in the range 61 ± 16 nm (16 nm is two standard deviations of the random error in contour length measurements as determined by measurements on free DNA as described above) were classified as looped complexes. RO complexes are defined as molecules that have one DNA arm length within 61 ± 16 nm and the other arm in the range of 108 ± 16 nm (108 nm is predicted based on the assumption that the sum of two DNA arm lengths should be approximately 169 nm, similar to the average image contour length measured for the 539- and 544-bp DNA molecules).

To permit objective statistical tests for differences in the population-averaged geometries of repressor–DNA complexes, the angle between the two DNA arms at their exit from the repressor, defined as the acute angle θ (Figure S1A) between two lines each tangent to the DNA arms at the two exit points, was measured by two independent observers. On average, images classified as non-looped repressor–operator complexes based on arm length criteria (Figure 1C, green squares) have a nearly colinear arm geometry (Figure S1A–S1C), with a mean arm exit angle (Figure S1A) of approximately 136°. This result is consistent with previous demonstrations that binding of repressor to a single operator site does not severely bend the DNA [76,77]. In contrast, complexes scored as looped (Figure 1C, red square) have a near orthogonal arm geometry on average, with mean exit angles of approximately 114° for looped complexes of O-158-O and approximately 100° for O-153-O (Figure S1D–S1M). Although from TPM experiments two looped species are observed in O-158-O, two peaks are not clearly resolved in the exit angle histogram (Figure S1H). The failure to resolve the peaks is perhaps not surprising; the equilibrium constants calculated from the TPM data predict that the equilibrium concentration of the short-tether loop is only approximately 13% that of the long-tether loop. Nevertheless, the population-average exit angle, measured by two independent observers, of O-158-O is significantly larger than that of O-153-O (*p* < 0.02 and *p* < 0.12 for observers A and B, respectively; unpublished data). Thus, AFM data support the conclusion from the TPM experiments that the population of looped complexes formed with O-158-O is structurally different from that formed with O-153-O.

### AFM image simulation.

The simulated images were generated using a program that allows the user to model objects on a surface using sphere swept lines (SSLs), which are cylinders capped on each end with a hemisphere [57]. The program then simulates an AFM image of the model by modeling the tip as a sphere swept cone, which is a cone capped with an 8-nm diameter hemisphere (comparable to the size of tip used in the AFM experiments) at the end, and convoluting the shape of the tip and modeled object using dilation and erosion methods. Lac repressor is modeled as an SSL with a diameter of 9 nm and a length of 11 nm (estimated from the crystal structures [20]), and the DNA is modeled as a chain made up of connected SSLs with flexible links, with each SSL being 4-nm long and 2 nm in diameter. The various conformation of the repressor–DNA complexes were modeled by manually wrapping or looping the flexible chain (DNA) around the 9 nm × 11 nm SSL (repressor). In all simulations, both the chain and the SSL lie on the surface. The length of the chain in the loop is approximately 50 nm, which is the distance between the two *lac* operator sites in the DNA used in the AFM experiments.

### Energetics of looped species.

The difference in standard free energy (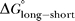 ) for O-158-O long-tether and short-tether looped complexes can be estimated from


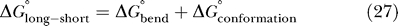


where 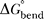 is the difference in the energy required to bend the DNA into a long-tether versus a short-tether loop and 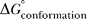 is the difference in conformation energy required to disrupt the protein-protein interactions in the V-shaped repressor upon loop formation. The value for 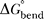 is calculated from


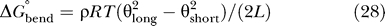


[78], where *ρ* is the bending persistence length of double-stranded DNA (∼154 bp, [79]), θ_long_ = 1.74π (2π − angle of the V arms of the repressor measured from the crystal structure) is the bend angle for the long-tether loop in Figure 4B, θ_shor*t*_ = π is the bend angle for the short-tether loop (Figure 4C) and *L* = 158 bp is the length of the DNA loop. Therefore, 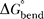 is 9.8 *RT*, where *R* is the gas constant and *T* is the absolute temperature, taken to be 298 °K in these calculations. Difference in conformation energy is calculated from:


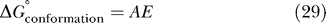


where A = 300 Å^2^ is the difference in solvent-exposed surface area [20] assuming short-tether loop formation requires opening up the V-shaped repressor to a fully extended structure and *E* = 66.9 J mol^−1^Å^−2^ [80]. Thus 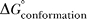 = ∼ −8.1 *RT* and 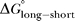 = 1.7 *RT*. The values of the latter determined from the measured equilibrium constant and from the global fit to the kinetic data are both −2.0 *RT*, in reasonable agreement with the estimate. The small discrepancy between the calculation and measurement may be attributable to either of two factors: First, the model does not include favorable sequence nonspecific interactions between the repressor core domain and the looped DNA segment [24] that may exist in the long-tether loop. Second, the two operator-binding sites are not precisely coplanar in the crystal structure, nor are they expected to be in the open structure; the DNA strain energies will thus be somewhat different from those in the estimate, which assumes a planar structure.

The change in free energy in introducing half a twist to DNA of length *L* is calculated from a simple elasticity model as:


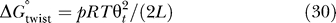


[78] where *p* is the torsional persistence length of double-stranded DNA (∼286 bp; [81]) and θ*_t_* is the torsional angle (π for half a twist) and *L* = 153 bp. Therefore, the energy needed for twisting 153-bp DNA half helical turn is 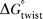 = 9.2 *RT*, which is equivalent to an equilibrium constant of approximately 10^4^.

### Loop model tether length estimation.

To estimate the long-tether loop tether length (Figure 4B), the unknown length of DNA that makes up each of the two overlapping arcs between the two DNA binding domains was approximated by the distance (corresponding to the length of approximately 32 bp of duplex DNA; see Figure 4B) between the center of one operator and the distal end of the other operator measured from the repressor–operator co-crystal structure [20]. Thus, the tether length for long-tether loop was estimated to be 354 bp, the sum of the two DNA arms (190 bp and 196 bp) minus 32 bp. For the proposed short-tether loop structure (Figure 4C), the arms do not overlap and the repressor is extended (both of which lengthen the tether), but the overall tether length is shorter. This is because the angle between the DNA arms as they exit the loop is roughly zero as opposed to the approximately 180° angle proposed in Figure 4B. The Figure 4C tether length is estimated to be the sum of the length of the extended repressor structure and the tether length contributed by the two DNA arms. Since the arm length is approximately the persistence length of the DNA, the arms will on average be bent by θ = ∼90°. Making the crude assumption that the effective tether length of the arm length *L* = 190 bp can be modeled as equivalent to a semicircular arc with radius *r* = *L /* θ = 121 bp, each DNA arm will contribute approximately 121 bp to the tether length of the short-tether loop. The length of the fully extended repressor conformation is approximately 54 bp, approximately twice the length of a repressor dimer estimated from the crystal structures. Therefore, the predicted tether length for the short-tether loop is 296 bp. This calculation is only an approximation; it assumes an arbitrary value for the arm exit angle and does not take into account sequence effects or protein or DNA dynamics.

## Supporting Information

Figure S1 DNA Arm Exit Angles in AFM Images Example AFM images (left) and exit angle histograms (right) of nonlooped repressor–operator complexes (A–C), O-158-O looped complexes (D–H), and O-153-O looped complexes (I–M). Images were classified as nonlooped or looped based on arm length criteria as in Figure 2. Exit angle is defined as the acute angle θ between two lines each tangent to the DNA arms at the two exit points from the repressor (A). Image sizes: 160 × 160 nm (A and B); 100 × 100 nm (D–G and I–L). All images were scanned from top to bottom.
(4.13 MB PDF)

Table S1 Kinetic Parameters of Raw Lifetime Distributions and Missed Event Corrections (37 KB DOC)

## Abbreviations

AFM: atomic force microscopy
TPM: tethered particle motion
WLC: worm-like chain
