# Supplementary figures and images for: Interconvertible Lac Repressor–DNA Loops Revealed by Single-Molecule Experiments
Source: PLoS Biol. 2008 Sep 30;6(9):e232. doi: 10.1371/journal.pbio.0060232 (PMC2553838; doi:10.1371/journal.pbio.0060232)

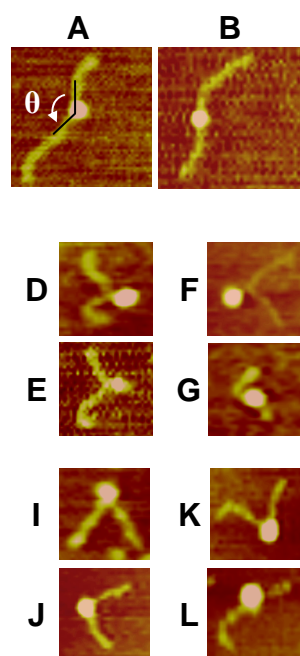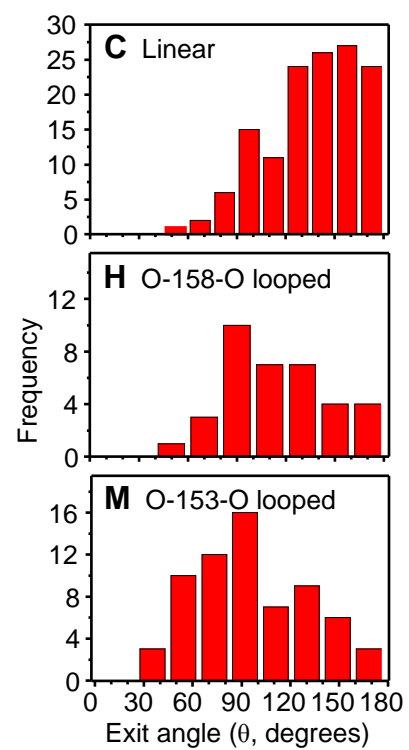

Supplement: Figure S1 — Example AFM images (left) and exit angle histograms (right) of nonlooped repressor–operator complexes (A–C), O-158-O looped complexes (D–H), and O-153-O looped complexes (I–M). Images were classified as nonlooped or looped based on arm length criteria as in Figure 2. Exit angle is defined as the acute angle θ between two lines each tangent to the DNA arms at the two exit points from the repressor (A). Image sizes: 160 × 160 nm (A and B); 100 × 100 nm (D–G and I–L). All images were scanned from top to bottom. (4.13 MB PDF) [file pbio.0060232.sg001.pdf]
