# Supplementary material for: Interconvertible Lac Repressor–DNA Loops Revealed by Single-Molecule Experiments
Source: PLoS Biol. 2008 Sep 30;6(9):e232. doi: 10.1371/journal.pbio.0060232 (PMC2553838; doi:10.1371/journal.pbio.0060232)
Supplement: Table S1 — (37 KB DOC) [file pbio.0060232.st001.doc]

**Table S1:** Kinetic parameters of raw lifetime distributions and missed event corrections

|  | τ (s)  *or*  τ_1_ (s), τ_2_ (s), *A* | *F* | *d* (s) | *D* (×10^3^ s) |
| --- | --- | --- | --- | --- |
| **O-153-O** |  |  |  |  |
| looped | 75 ± 3 | 0.12 | 75 ± 7 | 9 ± 1 |
| unlooped | 16 ± 2  193 ± 19  0.38 ± 0.02 | 0.21 | 125 ± 17 | 17 ± 3 |
| **O-158-O** |  |  |  |  |
| long-tether loop | 100 ± 3 | 0.10 | 100 ± 6 | 27 ± 2 |
| short-tether loop | 33.6 ± 0.3 | 0.25 | 33 ± 4 | 3.6 ± 0.5 |
| unlooped | 27 ± 1  97 ± 21  0.81 ± 0.05 | 0.27 | 40 ± 5 | 9 ± 1 |
